# How to Increase Sport Facility Users’ Intention to Use AI Fitness Services: Based on the Technology Adoption Model

**DOI:** 10.3390/ijerph192114453

**Published:** 2022-11-04

**Authors:** Ji-Hyoung Chin, Chanwook Do, Minjung Kim

**Affiliations:** 1 College of Education, Hankuk University of Foreign Studies, Seoul 02450, Korea; 2 Department of Kinesiology & Sport Management, College of Education & Human Development, Texas A&M University, College Station, TX 77843, USA

**Keywords:** technology adoption model, artificial intelligence, sport facility, perceived usefulness, perceived ease of use, exercise importance, attitude, intention to use AI service

## Abstract

Artificial intelligence (AI) has recently been introduced as a new way of analyzing and predicting sport consumer behavior. The goal of this study was to investigate the relationships among the perceived usefulness, perceived ease of use, the importance of exercise, attitudes towards use, and the behavioral intention to use AI services based on the technology adoption model. The authors recruited 408 participants who participated in an experiment designed to provide a deeper understanding of AI fitness services. After screening, the collected data were screened through assumption tests, and we conducted a confirmatory factor analysis and structural equation modeling to analyze research hypotheses. The results indicated that three types of consumer evaluations (i.e., perceived usefulness, perceived ease of use, and importance of exercise) positively influence their attitudes toward AI fitness services. In addition, the positive attitudes regarding AI services positively influenced the intention to use AI services. The results of this research contribute to our knowledge of the consumers’ attitudes and behaviors toward AI services in the sport industry based on the technology acceptance model. Furthermore, this study provided the empirical evidence critically needed to increase our understanding of AI in the sport industry and offered new insights into how sport facility managers can predict their consumers’ intention to use AI services.

## 1. Introduction

Artificial intelligence (AI) has already made a mark as a useful application in several fields (e.g., medicine, home appliances, entertainment, and vehicles) and is beginning to be employed in the sport industry. In the sport fields, many organizations have started using AI to understand sport consumer needs and provide better solutions [1]. The utilization of data generated through the application of AI technologies makes it easy for companies to (1) improve convenience, (2) enhance efficiency, and (3) better address individual needs [2,3]. Because of these advantages, companies are continuing their efforts to increase consumers’ familiarity with AI. For example, sports brands (e.g., Nike and Adidas) and big tech giants (e.g., Samsung and Apple) are investing in AI to assist consumers in the sports and healthcare sectors through the development of marketing strategies using AI. Therefore, communication with sport consumers using AI is becoming an important area for research [4].

AI can help companies to interpret data accurately and facilitate the data-driven learning of data to make decisions and promote and achieve their missions [5,6]. Further, AI can assist with measuring and analyzing action and movement, offering services including suggesting nourishment and, in this way, acting as an artificial trainer [7,8]. For instance, AI can design individual workout schedules and organize diet plans for athletes. After assisting with the design of individual workout and meal plans, AI can also supervise these plans. Additionally, an AI-facilitated, decision-making functionality could identify and offer the next workout level or the composition of a bespoke meal plan. In this way, AI-based fitness trackers have significant potential and could play a crucial role in the fitness industry [9].

As an example of AI usage, Sedol Lee, a formal South Korean professional Go player of the highest level, competed with AlphaGo, an artificial intelligence Go program developed by Google DeepMind [10]. Through the advancement of AI, it was able to predict that the development of science and technology will cause many changes across industries such as finance, law, medicine, and sports. Recently, AI has been often applied to sports fields [11]. For example, in tennis, AI helps coaches to establish strategies by analyzing player characteristics, physical strength, physical ability, and game patterns through a deep learning-based prediction algorithm [12]. Such functions are also valuable to predict the outcome of a baseball game [13]. The TRACAB system, for instance, was developed to detect signs of injury in advance by analyzing the image of the field (player posture, tools, referee, density, activity, etc.) and the behavior of people on the field [14].

Applying the Technology Acceptance Model (TAM), this study aimed to identify the factors that affect the attitudes of sports facility users toward AI, particularly regarding the importance of exercise and the perceived usefulness and ease of use of AI-based services. The TAM was based on the Theory of Reasoned Action (TRA) and is used for predicting and understanding individual behavior [15]. With respect to the TAM, the model consists of external variables, including perceived ease of use, perceived usefulness, and system use [10]. Furthermore, Venkatesh and Davis introduced an extended Technology Acceptance Model (extended TAM) which added additional various variables to the model that can be used to analyze AI, such as spontaneity, job relevance, image, and subjective norms [16]. The extended TAM revealed that perceived usefulness had a significant effect, and perceived usefulness and perceived ease of use were analyzed to measure intention to use [16]. In addition, the extended TAM revealed that playfulness and individual innovation affected perceived beliefs and that beliefs and self-efficacy, in turn, affected perceived usefulness and perceived ease of use [17]. 

Previous studies have revealed that the intention to use a specific type of technology largely depends on the characteristics of that technology [18]. Although several studies have attempted to identify the factors that influence the use of AI in various industries (e.g., tourism and health care), few studies have applied these effects to sport consumers. Therefore, this study seeks to expand the research area by using the extended TAM to analyze the intention of sport consumers to use AI services and to use the practical insights gained to suggest specific developments to sports practitioners. 

There have been limited empirical studies that apply TAM through the provision of AI services on behalf of humans in fitness centers. As the number of no-contact sport activities has been increased during, and after the COVID-19 pandemic, the application of AI services in physical activities and sports has become popular. In the era of the fourth industrial revolution, technological innovation and artificial intelligence-based technology have been expanding their application to various fields. In line with this trend, this study is expected to contribute to various sport industries by providing insights into analyzing the intention to use AI technology.

## 2. Literature Review

### 2.1. Perceived Usefulness

Perceived usefulness can be defined as “the degree to which a person believes that using a specific system will increase his or her job performance” [18]. Because perceived usefulness can enhance an individual’s current abilities, it is regarded as an important factor in evaluating technology. Furthermore, perceived usefulness has been found to have both direct and indirect effects on attitudes toward technology use, which can positively influence the behavioral intention to use the technology [16,19,20,21]. For example, athletes, coaches, and spectators who positively assess the perceived usefulness of an electronic competition scoring system are more likely to purchase the equipment [22]. Thus, perceived usefulness may influence both participants’ attitude and purchase intention.

Perceived usefulness can help to create personalized recommendations based on accumulated data [23]. AI systems can enhance perceived usefulness by answering inquiries and drawing conclusions based on collected data [5], thereby providing better service to consumers [24]. Thus, AI services can offer consumer-friendly recommendations, which can result in consumers regarding these services positively. Based on this argument, the first hypothesis is as follows:

**Hypothesis 1:**  *Perceived usefulness will positively influence attitudes toward AI services.*

### 2.2. Perceived Ease of Use

Perceived ease of use can be defined as the degree to which users feel that using new technologies and systems does not involve significant additional physical or mental effort [19]. Several TAM studies have demonstrated that perceived usefulness and ease of use are crucial predictors of the intention to accept or continue to use new technologies or services [25,26,27]. Thong et al. found that perceived ease of use positively affected perceived usefulness and had effects on the continued intention to use [28].

In addition, consumers’ perspectives on the ease of using a product can determine their attitudes toward a brand. To improve their evaluation, companies have tried to add advanced technology to their current systems. For example, Lee and Lee found that the perceived ease of use of AI robots at airports can influence the image of airports [29]. In the area of information technology, perceived ease of use is a critical variable in persuading users to accept or use a system [19,30]. We formulated Hypothesis 2:

**Hypothesis 2:**  *Perceived ease of use will positively influence attitudes toward AI services.*

### 2.3. Importance of Exercise

It is important to understand how to care for one’s health properly through exercise. For example, individuals who prefer to work out regularly have better mental health than those who do not [31]. In this sense, exercise can both help to ameliorate stress and enhance people’s abilities in terms of their job performance [32]. The recognition of the importance of exercise perceived by sports and exercise participants and students was an important variable in relation to exercise performance intention. According to Ban’s study [33], students taking after-school physical education courses in elementary school showed high levels of class satisfaction and exercise intention. Moreover, Ko analyzed the difference in school life adaptation according to elementary school children’s recognition of the importance of physical activity and reported that students who perceived it as important adapt well to school life [34]. The results of this study show that the recognition of the importance of physical activity affects the psychological satisfaction and behavioral results of individuals.

Because of the positive effects of exercise, many people regard exercise as a priority in their lives. To maintain this priority, it is necessary for people to use their time efficiently. Effective time management is imperative as people’s daily lives are very busy, and time management is a task for which AI can be an effective tool. Applying the appropriate AI tools to time management can effectively support the user [35]. In this sense, AI-facilitated exercise services based on customer data will be an effective tool for busy people who value exercise. Therefore, our third hypothesis is as follows:

**Hypothesis 3:**  *Individuals who value exercise will positively evaluate their attitude* *toward using AI services.*

### 2.4. Attitude and Intention to Use AI Services

Attitude towards utilization refers to the subjective evaluation of a user based on the latest AI technology or devices. In accordance with attitude models and decision theory, investigations have been conducted to characterize the effects of the application of the latest AI technology on user attitudes and decision-making [36]. When people perceive the advantage of using innovative technology, their attitude toward the use of innovative technology is influenced positively [37]. In this sense, it is imperative to identify people’s attitudes when new technologies are introduced.

Studies have highlighted the role of attitudes in predicting a learner’s intention to use technology [19,38]. Previous studies have shown that there was a significant relationship between attitudes and intention to use wearable devices, such as smartwatches [39]. Recently, research related to purchasing behavior related to sportswear to which innovative technology is applied has been conducted. In this study, the authors applied the TAM to smart clothing and concluded that perceived usefulness and perceived pleasure affect acceptance intention [37]. Additionally, perceived usefulness was found to influence acceptance intention by mediating attitude variables [40].

Behavioral intention includes word of mouth, repurchase, price sensitivity, and revisit, along with a comprehensive concept [41]. Behavioral intention has been used as a final variable in many studies and has been particularly emphasized as the core and most determinant factor of consumers [42]. A positive evaluation of new technologies can attract more new consumers. Based on this association, consumer intention has been used as a final variable in many studies and is highlighted as the most important determining factor in decision-making [43,44]. Recent researchers have classified behavioral intentions based on word of mouth, recommendations, and repeated purchases and visits [45,46]. Based on these previous studies, our study investigated the intentions of sports facility users to use AI services. In line with this argument, the fourth hypothesis is as follows:

**Hypothesis 4:**  *Attitudes to using AI services will positively influence the intention to use AI services.*

## 3. Materials and Methods

### 3.1. The Participants

**Figure 1:**
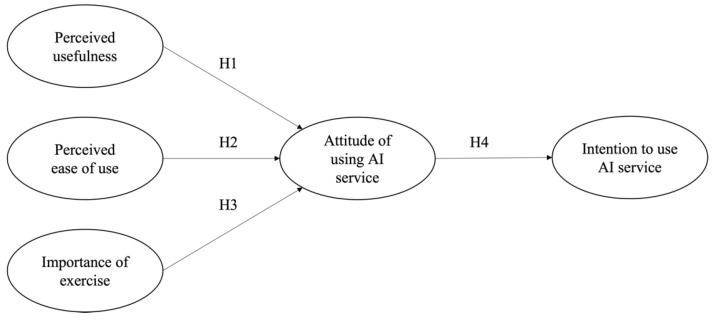
Hypothesized model of this study.

To test our hypothesized model (see Figure 1), we considered a place that can have a suitable environment to expose our experimental stimulants. Furthermore, if participants wanted to join this project virtually, we provided the Zoom link. Following suggestions from Belanche et al. and Mende et al., we contained the AI training service’s image and the stimulation description when participants read the material on the AI training system stimuli [47,48]. Based on these processes, we recruited 410 respondents who have an interest in using AI fitness services during their workouts using convenience sampling; however, two answers were excluded from this study because they reported incomplete answers in this survey.

Of the total sample (*n* = 408), 221 of the participants were women (54.2%) and 187 were men (45.8%); their average age was 26.13 (*SD* = 8.72). The respondents reported that their hobbies included spending time with friends (*n* = 95, 23.3%), watching and participating in sports (*n* = 64, 15.7%), watching TV (*n* = 55, 13.3%), enjoying the arts (*n* = 46, 11.3%), traveling (*n* = 36, 8.8%), and others (*n* = 112, 27.5%). They recorded their exercise frequency as zero (*n* = 28, 6.9%), once or twice a week (*n* = 136, 33.3%), three or five times a week (*n* = 202, 49.5%), or more than six times a week (*n* = 42, 10.3%). Among the total participants, 334 (81.9%) of the respondents answered that they were registered at a fitness center.

### 3.2. Experimental Stimulus

Prior to this experiment, an actual AI training device was used to prepare the experimental stimulus. The target of the stimuli to be manipulated was selected as a home AI exercise equipment from the US company T. The additional explanation presented to the stimulus was written in Korean based on the characteristics of the actual AI training apparatus. The AI training organization was named Healthy. The stimuli were presented with images and detailed information that consisted of three main parts. First, the appearance and characteristics of Healthy. The screen and component equipment, which are the external features of T’s AI training apparatus, are presented. In addition, it was described that Healthy analyzed the exercise behavior of the participants and that it can be used according to the time. Second, it is the principle of how Healthy works. As an AI training device, it was described that it operates based on infrared pulses, big data, and artificial intelligence 3D sensors. Finally, how to use Healthy was explained. 

Costs are presented along with specific usage methods. The cost is KRW 123,000, which includes KRW 75,000 for the fitness center and KRW 48,000 for the health service. The cost of the fitness center was measured based on data from the Ministry of Culture, Sports and Tourism, taking into account that the average cost of physical activity per person per month is KRW 78,000 [49]. The fee was set based on the monthly usage fee of AI training equipment commercialized in the United States. The average value of USD 41.2, which is the average value of USD 39 for company T, USD 39 for company M, USD 39 for company J, USD 49 for company T, and USD 40 for company N, was converted into KRW 48.000. 

### 3.3. Measure and Procedures

After reviewing relevant literature, this study was employed as the basis for a questionnaire (see Table 1) that has secured reliability and relevance, including the following sections: (a) perceived usefulness, (b) perceived ease of use, (c) importance of exercise, (d) attitude to use the AI service, (e) intention to use the AI service, and (f) demographic information (e.g., gender, age, and hobby). Except for demographic information, all questionnaires were adapted on a seven-point Likert scale ranging from 1 (strongly disagree) to 7 (strongly agree).

Based on this setting, we employed each of the four questionnaires of perceived usefulness and perceived ease of use from Belanche et al. [50]. Then, participants rated a series of exercise importance questions to evaluate their attitude toward exercise in their life [51]. Within this section, we omitted four questionnaires from a total of eight questions because they had similar meanings. After evaluating exercise importance, respondents answered their attitude toward using the AI service and their intention to use the AI service based on Belanche et al. [50]. Finally, they reported their demographic information.

## 4. Results

The authors checked representativeness of our data using SPSS version 22.0 and Amos version 20.0. First, a frequency analysis was conducted to understand the general characteristics of the participants. Second, reliability analysis of the measurement tool was checked by evaluating composite reliability values. It was found to exceed the standard value of 0.70 suggested by Nunnally and Bernstein; therefore, reliability was also secured [52], and confirmatory factor analysis (CFA) was performed to verify the validity of the questionnaire, including the comparative fit index (CFI), Tucker–Lewis index (TLI), root mean square error of approximation (RMSEA), and standardized root mean square residual (SRMR) [53]. In evaluating the full measurement model, the results showed a good model fit (χ^2^ = 119.68, *df* = 102, CFI = 0.98, TLI = 0.97, RMSEA = 0.05, SRMR = 0.04). The average variance extracted (AVE) values were calculated for all latent values which exceeded 0.50 [54]. 

Third, correlation analysis was performed to confirm the correlation and multicollinearity, incorporating the suggestion by Fornell and Lacker [54] and Hair et al. [55]. No correlation coefficient of 0.80 or higher was found, indicating that there is no problem with multicollinearity. Furthermore, this research considered using the solving common method basis because the survey was distributed in a random order [56]. Skewness and kurtosis were checked as conditions for confirming univariate stationarity. The skewness was less than 2.0 and kurtosis was less than 7.0 in all variables; therefore, a normal distribution was assumed [57,58], meaning that there was no abnormality in this research model (see Table 2).

**Figure 2:**
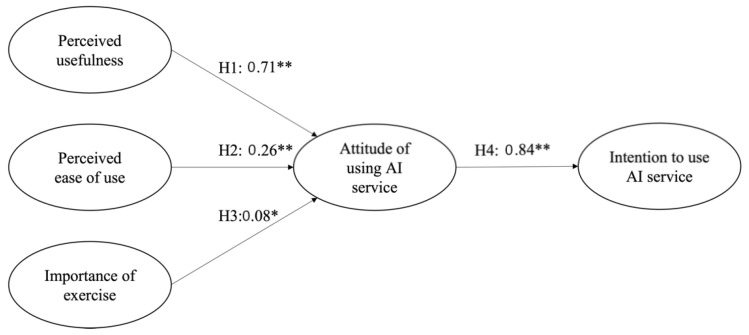
Hypotheses testing through structural equation modeling. Note: * *p* < 0.05, ** *p* < 0.001.

After identifying the acceptability of the full measurement model, structural equation modeling (SEM) was tested to verify our hypotheses. Our hypothesized model has a good fit to analyze the hypotheses (χ^2^ = 223.77, *df* = 106, CFI = 0.98, TLI = 0.97, RMSEA = 0.06, SRMR = 0.06). The coefficients of three paths for attitude to use the AI service and its predictors were positive and statistically significant: paths from perceived usefulness to attitude to use the AI service (H1: *β* = 0.71, t = 14.07, *p* < 0.001), perceived ease of use to attitude to use the AI service (H2: *β* = 0.26, t = 5.65, *p* < 0.001), and exercise importance to attitude to use the AI service (H3: *β* = 0.08, t = 2.28, *p* = 0.02). The paths from attitude to use the AI service and intention to use the AI service (H4: *β* = 0.84, t = 19.21, *p* < 0.001) were also statistically significant in the hypothesized directions (see Figure 2).

## 5. Discussion

In this study, we investigated how AI services influence consumers’ attitudes to use services, which potentially affects purchase intention based on the extended TAM. We found empirical evidence supporting the impact of attitudes regarding AI services on their purchase intention. We identified three predictors of attitudes regarding the use of AI services (i.e., perceived usefulness, perceived ease of use, and importance of exercise) and the attitudes influencing the intention to purchase AI services. 

Our first hypothesis was that perceived usefulness positively affects attitudes toward AI services (Hypothesis 1). Our results indicate a significant association between perceived usefulness and attitudes toward AI services, thus supporting Hypothesis 1. This result is consistent with previous research that reported that consumers recognized the useful function of new technology, which resulted in more positive attitudes towards the advanced technology [24,59,60]. Furthermore, the relationship between perceived ease of use and attitudes toward AI services was significant in the hypothesized direction, thus supporting Hypothesis 2. A positive evaluation of new technology depends on the ease of use levels [16]. The current result supports previous TAM research on attitudes toward AI services in which the levels of using AI services influenced consumers’ evaluation of the services, even though consumers had the experience of using the function [61]. 

Hypothesis 3 was supported, as our finding indicated that the perception of exercise importance influences attitudes toward the use of AI services. People who have thoughts about the importance of exercise in their life tend to positively evaluate the AI services that can recommend personalized workouts [62]. This result corresponds with previous research findings that found that individuals who regard exercise as important tend to have a positive attitude toward technologies that can promote their performance and prevent injuries [4,63]. Our study’s findings also verify the hypothesized relationship between attitudes toward AI services and AI services purchase intention (Hypothesis 4). A positive attitude about an event can stimulate certain behaviors [64]. When consumers have a positive memory of a brand’s product or service, they are likely to purchase the same items in the future [65]. In this sense, when sport consumers’ attitudes toward using AI services are positive, it is expected that their increased desire to purchase AI services will be associated with their favorable evaluation.

### 5.1. Theoretical Contributions and Practical Implications

This study has several implications for academics and practitioners. First, from a theoretical viewpoint, we expanded the range of the application of the extended TAM to the investigation of the acceptance and use of AI services by empirically examining the relationship between the predictors and outcomes in the context of the sport industry. Many studies on new technology have followed the extended TAM and a few scholars have investigated this new technology in the sport industry [59,66,67,68]. However, they have focused on augmented reality, virtual reality, and applications. This current research elucidated the effects of the application of AI services in the sport fitness industry based on the TAM.

Second, the significance of our current research lies in identifying the effect of AI services on the promotion of sport consumers’ purchase intentions. Previous AI services studies in the sport industry have focused on athlete performance analysis [62], forecasting injuries [69], and training [70]. Deviating from professional sport athletes’ applications, our research focused on sport consumers and how they perceive the usability of AI services and their attitudes toward the technology, as well as how those perception and attitudes influence their purchase intentions. By employing this approach in our research involving sport consumers, much-needed empirical evidence that deepens our understanding of the function of AI services, consumers’ exercise motivation, and consumer attitudes about AI services is gained.

The findings of our study offer practitioners insights into how to develop and implement the AI services and marketing strategies used by their organizations. First, fitness industry can expand its market share because of the new technology. If AI services can provide better suggestions for working out based on consumers’ accumulated data, customers may have a positive evaluation of this function of AI services. Nowadays, consumers recognize a unique value in their activities, and they may become loyal customers [71]. Furthermore, they can share their experience with their friends and colleagues [72]. Based on this evaluation, practitioners can effectively promote their services to gyms or communities where people enjoy sports, and the profits of these businesses and organizations can be expected to increase.

Second, our findings are expected to assist sports organizations in better understanding how people perceive the usefulness and ease of use of AI services. This new knowledge will help to demonstrate their utility and ease of use to users and hasten the development of services with integrated AI. For example, AI services that provide the ability to compare before and after exercise statistics will allow consumers to check on not only the results of their workouts but also their overall state of health as well. To increase consumer satisfaction, a simple interface and function and depth analysis based on user data are required. 

### 5.2. Limitations and Future Research

Even though this research study provides meaningful outcomes, it has certain limitations. The first is that future research should consider the use of an experimental study. Although the current research study conducted an online survey with a description of the functions of AI services, future scholars should use an experimental study and provide enough time to experience AI services to obtain precise data. Furthermore, when participants who value exercise importantly in their life experience the function of AI services, future study needs to generate an individual’s evaluation rather than those who have not been exposed to the AI [73,74]. The second limitation concerns the target population of this research. In this study, we considered our target population to include those who prefer to do individual exercises, such as, for example, weight training. In this sense, future research should be also expanded to include participants who enjoy team sports including soccer, baseball, and basketball. To do so, AI services will need to develop their functionality to include the analysis of players’ performance in these team sports.

The third limitation of this study is the negative function of AI. While AI has the potential to improve living and working and recreation conditions, it also raises significant questions about privacy, security, legality, and fairness [75,76]. As the machine can expose an individual’s private information to hackers, users who use AI services to improve their health need to consider this dangerous situation. In this sense, future studies can consider the various types of dangerous situations and how these concerns hinder the consumers’ intention of using AI services. Lastly, threats to human occupation have been widely discussed in the service sector [77,78]. Huang and Rust developed the AI job substitution theory to explain the process by which AI job substitution progresses from low to high intelligence [73]. They argued that the adoption of AI would make the service sector less demanding of employees’ analytical skills and emphasize rather intuitive and empathetic skills. Dhar argued that AI will create more jobs than it destroys [79]. However, Huang and Rust predict that AI will become a fundamental threat to service employment by leveraging its future potential through human–machine integration [73]. It is expected that research from various perspectives as well as variables measuring the advantages of AI service will be conducted.

## 6. Conclusions

To expand our understanding of AI in the sport industry, we investigated a model that measured attitudes toward AI services and the predictors of these attitudes that influence AI services on the purchase intention in respect of AI services. Perceived usefulness, perceived ease of use, and the importance of exercise were verified as predictors of positive attitudes that increase purchase intention. As an initial empirical investigation, this study provides a holistic and comprehensive analysis that is critical to a deeper understanding of the role AI services play in the sport industry. 

## Figures and Tables

**Table 1:** Finalized survey questionnaire: Mean, Standard Deviation (*SD*), Factor Loading (λ), composite reliability (CR), average variance extracted (AVE), and Reliability Coefficients (α).

Construct and Items	Mean	*SD*	λ	CR	AVE	α
**Perceived Usefulness**	5.79	1.00	-	0.88	0.70	0.87
Using AI service would improve my performance in exercising	5.73	1.09	0.87			
Using AI service would improve my productivity in exercising	5.94	1.09	0.80			
Using AI service would enhance my effectiveness in exercising	5.71	1.19	0.85			
**Perceived Ease of Use**	5.47	1.07	-	0.95	0.82	0.87
Learning to use AI service would be easy for me	5.26	1.38	0.70			
I would find it easy to manage my health using AI service	5.48	1.22	1.08			
It would be easy for me to become skillful at using AI service	5.39	1.29	0.89			
I would find AI service easy to use	5.74	1.15	0.92			
**Importance of Exercise**	4.78	1.32	-	0.87	0.63	0.87
Exercise is an important part of my life	5.72	1.33	0.84			
I get upset when I miss my exercise activities	4.10	1.64	0.66			
Exercise is among my top priorities	5.08	1.48	0.93			
I feel stressed when I do not find the time for exercise	4.24	1.73	0.71			
**Attitude of using AI service**	5.77	1.04	-	0.90	0.74	0.90
Using AI service for managing exercise seems like a good idea	5.78	1.14	0.88			
I like the idea of using AI service for managing exercise	5.88	1.12	0.82			
Using AI service for implementing my exercise seems like a wise idea	5.64	1.62	0.89			
**Intention to use AI service**	5.41	1.22	-	0.69	0.87	0.85
I intend to use AI service for managing my health	5.49	1.38	0.91			
Using AI service for exercise is something I would do	5.87	1.30	0.92			
Using AI service for implementing my exercise seems like a wise idea	4.88	1.49	0.64			

**Table 2:** Constructs, correlations, skewness (Skew), and kurtosis (Kurt).

	(1)	(2)	(3)	(4)	(5)	Skew.	Kurt.
(1) Perceived Usefulness	**0.84**					−0.73	0.27
(2) Perceived Ease of Use	0.58 *	**0.91**				−0.27	−0.77
(3) Importance of Exercise	0.24 *	0.29 *	**0.79**			−0.31	−0.39
(4) Attitude of using AI service	0.78 *	0.67 *	0.32 *	**0.86**		−0.64	−0.19
(5) Intention to use AI service	0.69 *	0.52 *	0.29 *	0.75 *	**0.93**	−0.81	0.69

Note: * *p* < 0.01.

## Data Availability

The data presented in this study are available on request from the corresponding author. The data are not publicly available due to privacy issues.
